# Examining the Complex Relationship Between Tuberculosis and Other Infectious Diseases in Children

**DOI:** 10.3389/fped.2019.00233

**Published:** 2019-06-25

**Authors:** Elizabeth Whittaker, Elisa López-Varela, Claire Broderick, James A. Seddon

**Affiliations:** ^1^Department of Paediatrics, Imperial College London, London, United Kingdom; ^2^Department of Paediatric Infectious Diseases, Imperial College Healthcare NHS Trust, St. Mary's Campus, London, United Kingdom; ^3^Desmond Tutu TB Centre, Department of Paediatrics and Child Health, Faculty of Medicine and Health Sciences, Stellenbosch University, Cape Town, South Africa

**Keywords:** tuberculosis, co-infection, risk, immunology, children, pediatric, mycobacteria

## Abstract

Millions of children are exposed to tuberculosis (TB) each year, many of which become infected with *Mycobacterium tuberculosis*. Most children can immunologically contain or eradicate the organism without pathology developing. However, in a minority, the organism overcomes the immunological constraints, proliferates and causes TB disease. Each year a million children develop TB disease, with a quarter dying. While it is known that young children and those with immunodeficiencies are at increased risk of progression from TB infection to TB disease, our understanding of risk factors for this transition is limited. The most immunologically disruptive process that can happen during childhood is infection with another pathogen and yet the impact of co-infections on TB risk is poorly investigated. Many diseases have overlapping geographical distributions to TB and affect similar patient populations. It is therefore likely that infection with viruses, bacteria, fungi and protozoa may impact on the risk of developing TB disease following exposure and infection, although disentangling correlation and causation is challenging. As vaccinations also disrupt immunological pathways, these may also impact on TB risk. In this article we describe the pediatric immune response to *M. tuberculosis* and then review the existing evidence of the impact of co-infection with other pathogens, as well as vaccination, on the host response to *M. tuberculosis*. We focus on the impact of other organisms on the risk of TB disease in children, in particularly evaluating if co-infections drive host immune responses in an age-dependent way. We finally propose priorities for future research in this field. An improved understanding of the impact of co-infections on TB could assist in TB control strategies, vaccine development (for TB vaccines or vaccines for other organisms), TB treatment approaches and TB diagnostics.

## Introduction

Each year millions of children are exposed to infectious cases of tuberculosis (TB) and estimates suggest that around 70 million children currently have TB infection globally (1). TB infection is a clinical state in which the child exhibits no symptoms or signs of disease, but, if tested, would have evidence of immunological sensitization to *Mycobacterium tuberculosis (M. tuberculosis)*, as detected through a tuberculin skin test (TST) or interferon (IFN) gamma release assay (IGRA). Each year about a million children develop TB disease (2) a clinical state characterized by symptoms, signs, radiological changes, and in some children, microbiological isolation of *M. tuberculosis*. Of these one million children, modeling studies suggest that 250,000 children die each year (3).

The majority of children with TB infection do not progress to TB disease. Most children are either able to eradicate the mycobacteria or contain them immunologically so that they do not cause pathology. Understanding which children are at high risk of disease progression, following infection, would be useful to better understand host-mycobacterial interactions which in turn could help with vaccine design, host directed therapies, as well as diagnostics that might assist in predicting which TB-infected children are at high risk of future disease. It would also be important to understand if there are factors that drive disease progression, as it may be possible to modify or eliminate these drivers if they are found to impact. Currently our understanding of why some children progress to disease while others do not is limited. Age, however, is crucially important.

**Figure 1:**
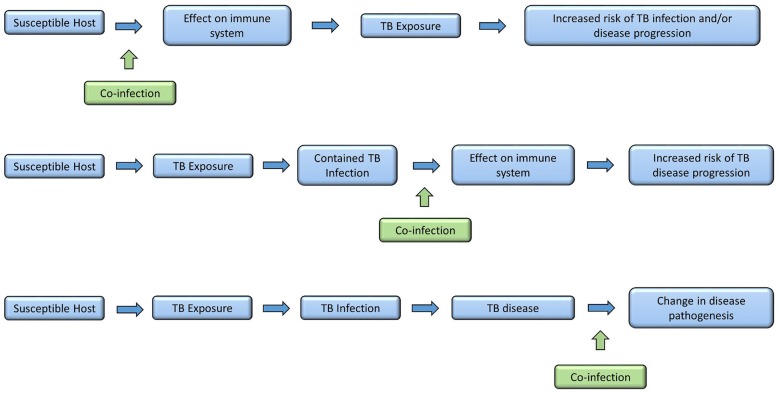
Times in disease spectrum and progression when co-infections may have an influence.

The risk of having a positive test of infection with *M. tuberculosis* increases in a fairly linear way with age, reflecting cumulative exposure (4). However, the risk of progressing from infection to disease is heavily age-dependent, with very young children at high risk of disease progression, the risk falling to a nadir in the pre-pubertal years, followed by a rise in risk as children enter adolescence (5). In addition the type of TB disease that children develop is age-dependent. The youngest children typically develop either intrathoracic lymph node disease (in which the mycobacteria are generally confined) or widely disseminated disease, including miliary TB and TB meningitis (reflecting poorly contained mycobacteria and unchecked proliferation). As children enter adolescence, the typical adult-type disease begins to manifest, with apical cavities and parenchymal breakdown, reflecting pathology largely caused by the host immune response. Although some societal and behavioral elements may influence these age-related changes in risk of disease progression and resulting disease phenotype, it is likely that to a large extent these result from age-related changes in the host immune system. These changes may be driven by a variety of environmental factors, including co-infection (Figure 1).

**Figure 2:**
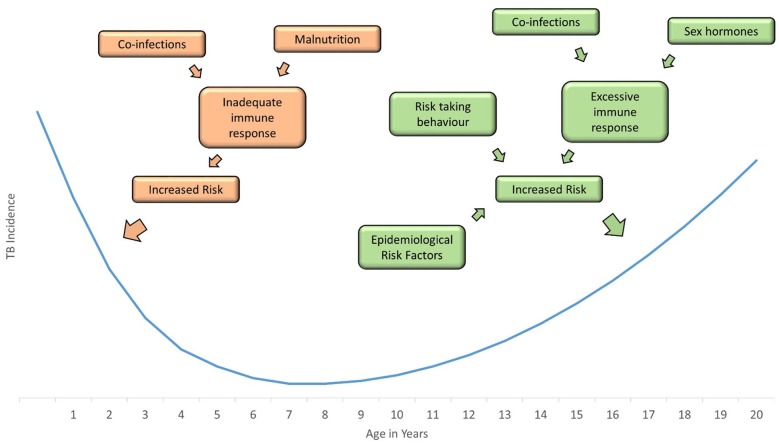
Incidence of childhood tuberculosis is greatest in infants and adolescents; a number of factors increase the risk in these age groups, including co-infections, hormones, behavior, and epidemiological risk factors.

**Figure 3:**
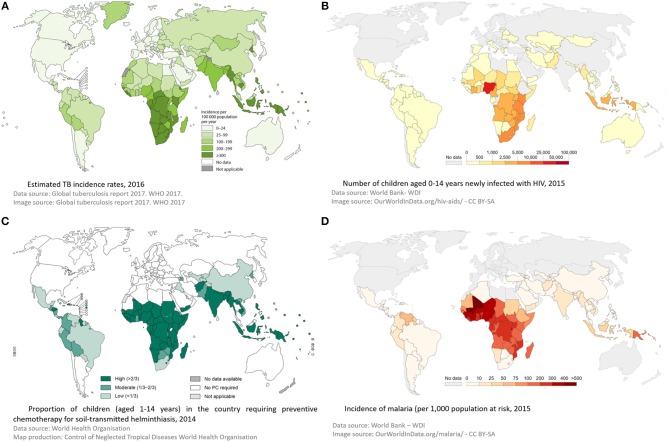
Global incidence of tuberculosis **(A)**, HIV **(B)**, Helminths **(C)**, and malaria **(D)**—demonstrating geographical overlap between these 4 infections, supporting a role for interplay between them. Panel **(A)** was reproduced from © World Health Organization (2). Panel **(B)** was reproduced from © World Bank -WDI (6). Panel **(C)** was reproduced from © World Health Organization 2015 (7). Panel **(D)** was reproduced from © World Bank -WDI (8). Data from WHO Malaria Report (9).

Through childhood, the events that have the most profound impact on a child's immune system are infections with pathogens, and to a lesser extent, vaccinations. Therefore, infection with a variety of pathogens could have a profound influence on the interaction between host immune system and *M. tuberculosis*. Concurrent infection prior to *M. tuberculosis* exposure could modify the host immune system so that following *M. tuberculosis* exposure there is an increased risk of subsequent TB infection or disease. Alternatively, a child may have well-contained TB infection and subsequent co-infection may then disrupt the carefully controlled immunological equilibrium, allowing *M. tuberculosis* to proliferate with TB disease progression resulting. Finally, a child may develop TB disease and then become co-infected with another organism. This may have an impact on the outcome of disease, in respect to resolution or severity (Figure 2). However, the relationship between infections with other organisms and *M. tuberculosis* have not been well-described. In this article we aim to bring together all the available evidence into one review. Where evidence is available, we have focused on evidence from co-infection with TB in children. If there are no data specifically in children, we present data on co-infection in adults, recognizing this is imperfect, but preferable to excluding those co-infections. Where appropriate, we have also considered the impact of other infections on a child's immune system and then discussed how this might impact on TB risk. We further acknowledge that for many organisms there is significant epidemiological overlap with TB in children, with challenges therefore in disentangling correlation and causation. We outline the geographical distribution of TB, HIV, helminth infections and malaria in Figure 3 as an example of this. For simplicity, where available, we provide specific evidence for pathogens that have been studied in greater detail, and for others, we group them together according to system, for example, respiratory viruses. Finally we suggest some key research priorities and possible study designs that might address them.

## Overview of TB Immunology in Children

The host immune response to *M. tuberculosis* involves both the innate and adaptive immune system, starting with antimicrobial peptides and neutrophils, followed by the interaction between the antigen presenting cells and the bacteria and granuloma formation, followed by a more targeted approach by CD4+ and CD8+ T cells (10–12).

**Figure 4:**
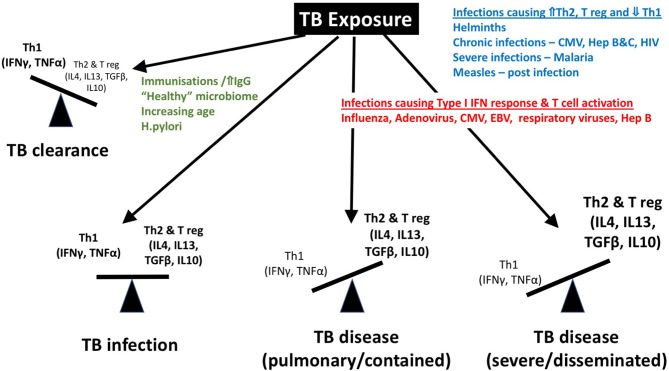
Although a “protective immune response” to tuberculosis remains elusive, a Th1 predominant response is associated with protection, while a Th2 and regulatory T cell predominance has been described in association with severe disease and dissemination. We propose that the balance of these immune responses is influenced by co-infections including helminths, CMV, hepatitis viral infections, malaria, measles, etc. IFN, interferon; IgG, Immunoglobulin G; TNF, tumor necrosis factor; T reg, regulatory T cell; IL, interleukin; TGF, tumor growth factor; HIV, human immunodeficiency virus; CMV, cytomegalovirus.

Should the bacilli be successful in traversing the physical and anatomical barriers encountered, *M. tuberculosis* bacilli are inhaled into the terminal alveoli where they are readily phagocytosed by resident alveolar macrophages and dendritic cells. This process results in activation of antimicrobial mechanisms, which serve to limit the growth of *M. tuberculosis* and recruit additional immune cells. Bacilli are processed and presented on the cell surface by antigen presenting cells that migrate to regional lymph nodes and present *M. tuberculosis* antigens to T cells. Secretion of a variety of cytokines, including IL-12 and IL-23, cause CD4+ T cells to proliferate and secrete cytokines, including IL-2, IFN-γ and tumor necrosis factor alpha (TNF-α), which further activate macrophages to become microbicidal if the encounter is successful. TNF-α increases the ability of macrophages to phagocytose and kill mycobacteria, stimulating apoptosis; this leads to increased presentation of mycobacterial antigens by dendritic cells (13). TNF-α also co-ordinates the inflammatory response through induction of IL-1, IL-6, and recruitment of macrophages, Natural Killer (NK), γδ and CD8+ T cells promoting their activation (14). Absence of TNF-α is associated with progression to severe TB disease, as seen following treatment with anti-TNF monoclonal antibodies for autoimmune conditions (15, 16). Yet, excessive TNF-α promotes immunopathology by interfering with cell death processes and induction of a hyper-inflammatory milieu. As with so many factors in the immune response to TB, balance is critical. A neutrophil-driven, IFN-inducible transcript signature in adult whole blood was recently identified that correlated with clinical severity, (17) and neutrophilia has been associated with poorer prognosis. Additionally, T cell activation, as measured by HLA DR+ expression and production of cytokines such as IFN-γ, IL-1β, and TNF-α has been shown to be associated with TB disease (Figure 4) (14, 16, 18, 19).

A variety of age-related immune differences have previously been described in the host response to mycobacteria (11, 12). Most notably, young children have fewer antigen presenting cells, with reduced functional responses, including phagocytosis and recruitment capacity, compared to older children and adults. These functional impairments lead to poor T-cell priming and consequently impaired immunity to *M. tuberculosis*. The role of non-conventional T cells, such as γδ T cells, NK T cells, Th17 and regulatory T cells, which link or modify the innate-adaptive T cell interaction have recently been explored (20, 21) they are noted to be increased in younger children with TB disease compared to similarly aged healthy controls (20).

Other components of the innate immune response which are likely to be affected by co-infection, and are recognized to be different in neonates and early infancy, include levels of innate defense molecules such as collectins; maturation of Toll-like-receptors (TLRs) and neutrophils.

The immune mechanisms involved in these processes are poorly defined and the dynamic balance that exists between bacterial persistence and host defense, which in children tips in favor of the mycobacteria more than in adults, can be influenced by several factors, including, we hypothesize, age and co-infection with various pathogens (Figure 4).

In longitudinal studies, many acquired immuno-suppressive conditions are known to disrupt this balance and increase the risk of TB disease, including HIV infection, malnutrition, vitamin D deficiency, diabetes, and anti-TNF-α therapy. Here we aim to explore the evidence that co-infections play a role in disrupting this balance (Table 1).

**Table 1:** A summary of (1) the predominant immune response associated with a variety of pathogens in children and (2) the known impact of that pathogen on tuberculosis pathogenesis.

**Pathogen**	**Impact on pediatric immune system**	**Potential impact on TB pathogenesis**
TB	Th1, IFNγ, IFNα, TNFα, IL12, IL23, IL17, IL2	Severe disease- increased regulatory T cells, suppressed Th1, excessive neutrophils
HIV	Decreased Th1, Low IFNγ, increased apoptosis due to IL10 and XS TNFα. Specific depletion of CD27 activated mycobacterial specific CD4+ T cells	Increased pathogenesis, increased risk of infection, disease, and severe disseminated disease
CMV	Adults—clonal expansion CMV specific CD4+ and CD8+ T cells Children—suppressed IFNγ producing CD4+ T cells compared to adults Strong type I IFN response CMV IFNγ ELISpot response associated with T cell activation (HLA-DR)	CMV specific IFNγ producing T cells and T cell activation associated with increased risk of TB disease progression. Both CMV and TB induce Type I IFN signature
EBV	CD8+ T cell response predominates, strong type I IFN response	Unknown
HHV6/HHV7/HSV	Increased NK cells, CD4, and CD8 clonal expansion	Unknown in humans Herpes virus latency in mice protective against bacterial infection due to persistent IFN signature
Hepatitis B and C	Hep B—CD8+ cytotoxic T cells, Type I interferon, NK cells. Chronic infection—increased regulatory T cells, raised IL10, suppressed IFNα, IFNγ Hep C—virus infected T cells lose cytotoxicity and ability to produce IL2, TNFα, IFNγ, increased regulatory T cells, and IL10	Unexplored, but chronic infection with either Hepatitis B or C is associated with higher regulatory T cells and IL10, lower IFNγ, IFNα which may increase susceptibility to TB in children
Microbiome and gut-lung axis	Modulates innate immune responses through TLR stimulation—*Helicobacter pylori* associated with increased TB specific Th1 responses	Hypothesized to be protective through persistent Th1 stimulation Unknown if different bacteria cause differential stimulation of Th1 leading to changes in susceptibility
Adenovirus	Primary Adenovirus infection suppresses IFNγ, while secondary reactivation leads to increased IFNγ, as well as HLADR+ and Ki67+ T cells	Adenovirus primary infection may result in immunosuppression, while secondary infection may result in T cell activation (as measured by HLA-DR), both associated with increased risk of TB disease progression
Respiratory viruses	Influenza—induces type I IFN signaling pathway Downregulates TLR stimulation, decreased neutrophil recruitment. Other respiratory viruses—RSV, Metapneumovirus, Adenovirus, corona virus, etc., all induce type I IFN, so likely similar pathogenesis	Influenza—seasonal association with TB disease possibly due to local lung damage Type I IFN associated with progression to TB disease, also suppressed TLR responses and neutrophils In mice influenza is associated with increased mycobacterial growth
Measles	Increased IL6, IL1b, TNFα, IL8, decreased IL12, IFNα. Th2 responses and increased regulatory T cells predominate for several weeks the acute infection	Measles infection associated with transient immunosuppression for weeks/months—recorded increased incidence TB disease in children, in particular TB meningitis
Fungi	Similar immune responses—Th1, poor TLR stimulation, granuloma formation	Co-infection, or concurrent infection likely due to lung damage rather than immune impact on susceptibility
Respiratory bacteria	TLR and NLR stimulation by bacteria stimulates phagocytes and a subsequent innate immune response including natural killer cells, pro-inflammatory cytokines, and adaptive immunity via T and B cells	TB often complicated by bacterial co-infection, or TB follows bacterial damaged lungs. Co-infection associated with increased mortality Immune response to bacteria likely to be protective against TB
Other non-TB bacteria	Intracellular bacterial infections elicit similar cell mediated response (CD4+ CD8+ and T cell activation)	Similar immune responses may represent protection. Co-infection not commonly reported
NTMs	Th1 immunity including CD4+ T cells responses and neutrophils all essential for protection against NTM infections	TB disease and lung damage likely to predispose to NTM, rather than the other way around. Concurrent infection seen
Helminths	IgE, IgG4, Th2 cytokines (IL4, IL9, IL13), Regulatory T cells and cytokines (TGFβ, IL10)	Shift away from Th1 likely to contribute to immune susceptibility and progression to disease. Mixed evidence to date
Malaria	Pro-inflammatory cytokines (IL1β, IL6, IL12, TNFα, IFNγ) stimulated by infection. Severe disease associated with decreased levels of CD4+ cells and associated immunosuppression *In vitro*, malarial parasites decrease the humoral and cellular response to TB	Severe malaria disease associated with Th2, IL10, and low CD4/immunosuppression which may increase susceptibility to TB disease Co-infection associated with increased Th2 responses and IL10. Both TB and malaria associated with MMP9 induction
Other protozoa	Suppressed Th1 responses (low IL12)	Likely to impact TB immune responses, limited evidence
Routine vaccinations	Increased total IgG levels	Protective against TB infection

*IFN, interferon; IgG, Immunoglobulin G; TNF, tumor necrosis factor; IL, interleukin; TGF, tumor growth factor; HIV, human immunodeficiency virus; CMV, cytomegalovirus; EBV, Epstein Barr Virus; HLA-DR, human leukocyte antigen-DR; HHV, human herpes virus; NK, natural killer; TLR, Toll like receptors; RSV, respiratory syncytiovirus; NLR, nod- like receptor*.

## TB-HIV Co-infection

The “deadly duet” of TB-HIV co-infection has been extensively studied and World Health Organization (WHO) guidelines recommend all children diagnosed with TB should be screened for HIV and conversely, children newly diagnosed with HIV should be screened for TB (22). Dodd et al. in a systematic review of the impact of HIV on TB in children, reported that HIV infection increases the incidence of TB in children by a factor of around eight, increasing with degree of immunosuppression (22). Combination antiretroviral therapy (cART) can restore immune function and has enormously reduced morbidity and mortality among HIV-infected children and is strongly protective against TB, reducing the risk by 70%. However, it takes 2 years for the full potential of protection against TB to be realized. The impact of age on efficacy of antiretroviral therapy is complex, as early treatment initiation, at a better baseline immune status, leads to better immune reconstitution (23). Initiation of cART in children and adults can be complicated by immune reconstitution inflammatory syndrome (IRIS), thought to be a dysregulated immune response to a pathogen, most commonly TB. There is a paucity of data on the epidemiology, risk factors, management, and outcome of TB-IRIS in children (24).

Studies in adults have demonstrated that HIV infection increases susceptibility to TB primarily through decreased numbers of CD4+ T cells and impaired function of CD4+ T cells, in particular in their response to phagocytes (25). As the increased risk of TB is present in HIV-infected individuals prior to significant T cell depletion, this suggests that HIV may alter cellular responses to *M. tuberculosis* infection. Studies in TB-HIV co-infected adults to characterize functional defects in CD4+ T-cells has increased our understanding of the role of these cells in the immune response to *M. tuberculosis*. HIV preferentially infects and depletes mycobacterial specific T-cells, most likely due to their activated, CD27 expressing, IL-2 producing state (26). HIV infects other cells including macrophages, dendritic cells and neutrophils, influencing cytokine production, and T cell interactions, which may impact on susceptibility to TB infection, progression to and severity of disease. HIV-infected macrophages act as a reservoir for the virus, leading to TNF-α-induced suppression of apoptosis, thus avoiding immune-mediated clearance by the host (27). HIV1 *nef* and *M. tuberculosis* antigen *Rv3416* synergistically contributes to anti-apoptotic signaling in macrophages (28, 29). Furthermore, IL-10, produced by macrophages and regulatory T cells, also decreases apoptosis. Plasma IL-10 levels are higher in TB-HIV co-infected adults with pulmonary disease compared to those with HIV infection alone, or those with HIV and TB infection (30). These findings have not been explored in children, although HIV-uninfected children with TB disease have been found to have higher levels of regulatory T cells and IL-10 than healthy controls or children with TB infection (20). Myeloid derived suppressor cells (MDSC), an innate immune cell population known to downregulate T cell proliferation, are increased in adults and children with TB infection and disease. Recently, high levels have also been identified in HIV exposed uninfected infants (HEU), who are known to have increased susceptibility to TB, suggesting a potential mechanism for TB susceptibility. Interestingly HIV infected children on HAART did not have increased levels of MDSC, suggesting that HIV viraemia triggers these regulatory innate immune cells (31). Understanding how HIV increases TB risk in children presents a key research priority.

## Herpes Viruses

The herpes viruses that cause pathology in humans include herpes simplex (HSV) 1 and 2, Epstein-Barr virus (EBV), cytomegalovirus (CMV), varicella zoster virus (VZV), and the human herpes viruses (HHV)6-8. Viruses from this family have sophisticated mechanisms for evading the host immune system and consequently establish long-term infections, fluctuating between periods of active disease and periods of inactivity or latency. This is particularly pertinent in children infected in infancy.

CMV infection of the mother during pregnancy can be associated with congenital infection of the fetus, leading to fetal loss and, in surviving infants, neurodevelopmental delay and hearing loss (32). CMV infection has also been well-studied in individuals with various forms of immunosuppression, such as HIV infection, primary immunodeficiencies or post bone marrow transplant (33–35). In these populations it can cause a variety of pathologies, including pneumonitis, retinitis, gastroenteritis and central nervous system dysfunction, among others. CMV infection has also been well-studied in older individuals, causing increased cardiovascular risk and immunosenescence (36, 37). The impact of CMV infection in immunocompetent children, however, is poorly understood.

This is a significant gap in our understanding, as CMV is one of the most prevalent and immunogenic viruses that infect children. In low resource settings, the vast majority acquire CMV in early childhood (38, 39). CMV is acquired from secretions and, in immunocompetent children, usually causes either an asymptomatic viraemia, or viraemia associated with infectious mononucleosis-like symptoms (40). IgG seropositivity usually develops as a consequence of infection. Reactivation, with viraemia and/or symptoms can occur at any point later in life. CMV infection can lead to clonal expansion of differentiated CMV-specific CD4+ and CD8+ T cells, an effect that can last for years (41). Children with asymptomatic CMV infection have been shown to have markedly fewer CMV-specific CD4+ T cells that produced IFN-γ, compared to adults with asymptomatic CMV infection, an effect that lasted for over a year (42). Regarding the relationship with TB, CMV elicits a strong type I IFN response, (43) a response demonstrated to be associated with TB disease (44). In a trial of the MVA85A TB vaccine in infants, Fletcher and colleagues found a significant correlation between CMV IFN-γ ELISpot response at baseline and T-cell activation, in turn associated with future TB disease progression (18). Further analysis of this cohort has demonstrated that a CMV-specific IFN-γ response was associated with increased risk of developing TB disease (45). Groups have also postulated that there is significant overlap in epidemiology between CMV and TB, with new infections of CMV common in early childhood and again in adolescence—potentially impacting on the changes in incidence seen with the TB epidemic (46).

EBV is also a ubiquitous virus, with estimates suggesting that nearly 90% of the global population is infected (47). In resource limited settings, the majority of primary infection is in early childhood, likely as a result of exposure to saliva and resulting in asymptomatic infection (48). Infection in adolescence results in extensive expansion of activated EBV-specific CD8+ T-cells, (49) with an expression profile that suggests uncontrolled inflammation and a strong type I IFN response. Primary infection in young children, however, seems to elicit a virus-specific CD8+ T-cell response that is able to contain the virus without over-expansion (50). Given the overlapping age profiles and the immunological effects of EBV infection, a relationship with TB is very possible.

The other herpes viruses are also very common. Most individuals have serological evidence of exposure to both herpes simplex viruses by adulthood, with more rapid acquisition in resource limited settings (51). Prior to vaccination strategies for varicella, most children had developed chicken pox during childhood (52). Most children are infected with HHV6 by the time they are 2 years of age, many developing a symptomatic illness at the time of primary infection (53). Positive HHV7 serology is almost universal by adulthood (54) and HHV8 is common in low resource settings, mainly acquired during childhood (55). No studies have directly evaluated the relationship between these other herpes viruses and TB. However, the herpes viruses have a marked effect on the host immune system and, dependent on the stage of herpes infection (acute infection, latency, or reactivation) and the timing in relation to TB pathogenesis, could impact on TB risk. Herpes viruses can “arm” NK cells (56) and even during periods of latency, herpes viruses maintain large populations of functional CD4+ and CD8+ T cells. In mouse models herpes virus latency leads to persistent production of IFN-γ and systemic activation of macrophages protects mice from infection with the bacterial pathogens *Listeria monocytogenes* and *Yersinia pestis* (57). It is likely that herpes virus infection impacts on the human response to multiple other pathogens, including *M. tuberculosis*, however whether this leads to disease or is protective requires further research (58–61).

## Hepatitis B and C

Hepatitis B and C viruses (HBV, HCV) are important infective causes of chronic hepatitis and cirrhosis. Up to 30% of the world's population show serological evidence of past or current HBV infection and an estimated 257 million people are living with HBV infection (HBV surface antigen positive). Prevalence is highest in the WHO Africa region (6.1%) and the WHO Western Pacific region (6.2%). In highly endemic areas, HBV is acquired perinatally through vertical transmission from the mother to neonate, or during early childhood. In lower endemic settings, typically older susceptible children may become infected through exposure to contaminated blood, through sexual transmission or intravenous drug use. The outcome of acute HBV infection is largely age-dependent with 80–90% of infants and 30–50% of children aged 1–6 years old developing chronic infection, compared to just 5% of adults. Since the introduction of the HBV vaccine, the estimated global prevalence of chronic HBV in children aged 5 years and under has fallen from 4.7 to 1.3% in 2015, though it remains 3% in Africa where TB co-infection is likely to be common (62). Studies have found the prevalence of HBV infection to be higher amongst TB patients than the general population (63–67).

The immune mechanisms of viral clearance are unknown, but both humoral and cellular immune responses are involved. Clearance occurs following the generation of antibodies against viral envelope antigens that clear virus particles, with CD4+ T helper cell interaction playing a vital role. CD8+ cytotoxic T cells eliminate infected cells directly (68). Chronic infection is characterized by a relative immunosuppressive state, perhaps induced directly by the virus. This relative immunosuppressed state in chronic infection in adults is characterized by higher numbers of CD4+CD25+ FOXP3+ regulatory T cell, increased levels of IL-10, and impaired IFN-α and IFN-γ production. Healthy uninfected infants are known to have diminished IFN-γ responses and increased numbers of regulatory T cells—an immune state implicated both in susceptibility to TB infection, TB disease progression and chronicity of hepatitis B infection (69). Further work to determine whether chronic hepatitis B infection in infancy in association with this described immune phenotype is implicated in TB disease progression is required.

Approximately 1% of the global population (71 million) is HCV-infected with an estimated 1.75 million new infections occurring in 2015. An estimated 5 million children under 15 years have chronic viraemic infection. Mother-to-child vertical transmission occurs in 6% of HCV-affected pregnancies, and accounts for up to 60% of pediatric cases of HCV. Among vertically-infected children, up to 80% develop chronic infection (70). Several studies report on the prevalence of anti-HCV antibodies in TB patients, but very few report the prevalence of detectable HCV RNA and thus active infection (63, 67, 71, 72).

Similarly to hepatitis B, the mechanisms of viral clearance are not fully understood, but CD4+ and CD8+ T cell responses seem to be crucial. Those who clear the virus have better T cell proliferation and IL-2, IFN-γ, and TNF-α production than those who develop chronic infection (73). Neutralizing antibodies are not required to clear HCV as demonstrated in hypogammaglobulinaemic patients (74). Like chronic HBV infection, a downregulation of virus-specific T cell responses is observed in chronic HCV infection, with progressive depletion and functional exhaustion of virus-specific CD4+ and CD8+ T cells. Virus-specific T cells lose their cytotoxicity and their ability to produce IL-2, TNF-α and IFN, though production is less impaired than seen in chronic HBV (74). Increased numbers of FOXP3+ regulatory T cells are observed in the blood and the liver (75) and levels of the immunosuppressive cytokine IL-10 are also increased (76). NK cells are activated in chronic HCV infection, though IL-10 and IFN-α suppress NK-production of IFN-γ and TNF-α (74).

Little is known about the effects of HBV and HCV co-infection on TB in children, but given the effects of these viruses on immune responses and their widespread distributions globally, an interaction seems plausible.

## Measles

The global burden of measles remains high with an estimated 7 million cases and 89,780 measles-related deaths in 2016, most of which occurred in children under five and in low income countries with poor health systems. Severe disease is most common in poorly nourished young children, particularly those with immunosuppression, or vitamin A deficiency (77).

Young infants are protected against measles by passively acquired maternal anti-measles virus IgG. Maternal antibodies are generally higher in women who had natural measles infection rather than those with vaccine-induced immunity, so children of vaccinated women tend to become susceptible at a younger age (78). The average age of measles cases is dependent on the rate of decline of protective maternal antibodies, the age at which children are vaccinated against measles, and the rate of contact between susceptible and infectious individuals. In densely populated urban centers with poor vaccination coverage, measles is a disease of infants and young children. As measles vaccine coverage increases, the incidence of measles reduces and there is a shift toward cases being in adolescents and adults (79). This may be of relevance in pediatric TB-measles co-infection as it means that measles is increasingly likely to affect older children with TB infection rather than very young children at risk of primary TB.

Measles virus initially infects lymphocytes, dendritic cells, and alveolar macrophages in the respiratory tract, stimulating production of pro-inflammatory cytokines IL6 and IL8, and suppression of IL12 *in vitro* (80–84). *In vivo* studies of children with measles demonstrate increased production of pro-inflammatory cytokines IL-1β, TNFα, IL8 (85). The role of type I IFN is less clear with *in vitro* measles infection leading to variable IFN responses, depending on cell type (86–88). *In vivo*, expression of IFN-stimulated genes is not increased in the peripheral blood mononuclear cells (PBMCs) of children with measles, though these samples tend to be taken at the time of the rash, when measles is recognized, and therefore may miss IFN production if it occurs earlier in infection (85, 89).

CD8+ T cells are important for viraemic clearance which occurs within a few days of the onset of the rash (90). After viraemic clearance, numbers of circulating activated CD8+ T cells and plasma levels of IFN-γ fall rapidly. Circulating activated CD4+ T cells reduce in number much more slowly, possibly due to the continued presence of measles virus-infected cells (91). Early in the immune response, CD4+ Th1 responses predominate, with IL-2 and IFN-γ production. As the virus is cleared and CD8+ cells and IFN-γ levels decline, there is a switch to CD4+ Th2 cell responses with production of IL-4, IL-10, and IL-13 which lasts for several weeks. Regulatory T cells are also prominent (92). This is thought to promote B cell maturation and establishment of humoral memory.

Measles infection is associated with transient immunosuppression lasting weeks to months, though the underlying mechanisms are incompletely understood (90). Secondary infections, particularly in the respiratory and gastrointestinal tracts, are important causes of measles-associated mortality (93, 94). Transient lymphopenia, (95) disappearance of tuberculin skin reactivity, (96) impaired lymphocyte proliferative responses (97), and impaired dendritic functions (98–100) have all been described. The Th2 and regulatory T cell predominance also depresses macrophage activation and suppresses lymphoproliferation and induction of Th1 responses in response to other pathogens, including *M. tuberculosis*.

The clinical effect of measles co-infection on TB has been studied since at least the early 20th century with the observation that the TST becomes transiently negative during measles infection, (101, 102) returning to previous levels of reactivity 2–4 weeks later. Several reports from measles epidemics have suggested that measles co-infection has a deleterious effect on TB containment in both children and adults. (103, 104) TB typically appears 2 months after measles infection and an American hospital noted that almost 10% of their pediatric TB meningitis cases experienced their first symptoms while they were convalescing from measles (105). In the 1960s, increased TB relapses in those with wild measles compared to those with vaccine measles or no measles, were reported. The relapses were observed 2 weeks to 3 months after the measles, at a similar time to their regaining tuberculin skin reactivity (105). However, a later review by Flick reported deficiencies with many of these studies, concluding there was inadequate data to support a significant interaction (106). More recently, a study of the 2000–2001 measles outbreak in Korea estimated the overall incidence of TB cases following measles to be lower than the general population (107). An Iraqi study reported increased anti-measles IgG antibody titres in adults with TB compared to controls, the authors suggesting that immunosuppression induced by recent measles infection or reactivation may have triggered reactivation of TB infection (108). The impact of measles on TB in children, is therefore far from clear and further well-designed studies, both of the impact of measles infection and measles vaccination, are warranted.

## Human Adenovirus

Human adenoviruses are a common cause of childhood infections worldwide. Their ability to infect many cell types makes them useful vectors for gene delivery (109) and their immunogenic properties mean they are also used as vaccine vectors (110). The majority of primary infections occur during the first 5 years of life. In children, adenoviral infections account for up to 15% of upper respiratory tract infections and about 5% of lower respiratory tract infections (LRTI) (111). Latent infection may follow primary infection with latency described in tonsillar tissue, T lymphocytes and lung epithelial cells. (112–115). Reactivation is important in the severely immunosuppressed (116, 117).

The immunological effects of adenovirus infection are complex (118) and may vary depending on past exposures. Different adenovirus species share a common hexon protein which is a key T cell target (119). Exposure to one human adenovirus therefore generates cytotoxic CD4+ and CD8+ T cells which cross-react with multiple adenovirus species. This is thought to contribute toward broad immunity in adults (120). In rhesus macaques, T cell and cytokine responses to human adenoviruses have been shown to vary with repeated exposures, with increased IFN-γ mRNA expression in PBMCs and increased frequencies of Ki67+, HLADR+, and CD95+/CCR5+ CD4+ T cells in blood recorded after the secondary, but not the primary exposure, mRNA expression of CCL20, TNF-α, and IL-1β in PBMCs was reduced after primary exposure and further suppressed on repeat exposures (121). Given the frequency of adenovirus exposure in young children, cross-reactivity between different virus types, and potential for latency and reactivation, an interaction between TB and adenovirus could be important and further studies are warranted.

## Respiratory Viruses

### Influenza

The global burden of influenza disease in young children is high, with an estimated 90 million new cases of influenza and 20 million cases of influenza-associated acute LRTI in children <5 years old each year. Of the estimated annual 28,000-111,500 pediatric deaths attributable to influenza LRTIs, 99% occur in developing countries (122).

Epidemiological and modeling studies of previous influenza pandemics suggest an interaction between influenza and TB. An analysis of Swiss historical data found that TB-associated mortality increased during the 1889 and 1918 influenza pandemics and declined thereafter (123). This selective mortality of TB patients is suggested to have contributed to the subsequent decline in TB mortality observed in USA, Japan and the Netherlands, by killing people with TB and reducing transmission (124–126). Studies of more recent influenza outbreaks have found that patients co-infected with TB are at greater risk of dying from influenza than those without TB, even when adjusted for HIV status (127, 128). Amongst patients with TB, those with influenza co-infection have an increased risk of death compared to those without. However, the evidence is mixed (129) and these studies cannot ascertain the mechanisms underlying these observations. It has been hypothesized that lung pathology due to respiratory viral infections predisposes to TB pathogenesis.

Evidence suggests that TB-influenza interactions in children may be complex. In South Africa, where there is seasonal variability in pediatric TB hospitalisations, a temporal association has been observed in hospitalized children between cases of influenza, pulmonary TB, and invasive pneumococcal disease (IPD) (130). A seasonal pattern of influenza activity is followed by a peak in pulmonary TB cases 2–3 months later and IPD 3 months later. This 2–3-month window between influenza and TB peaks corresponds to the time it takes for a young TB-exposed child to develop primary TB, and for TB infection to progress to TB disease. The authors speculated that during influenza outbreaks, young children living with adults with infectious pulmonary TB could be at enhanced risk of TB exposure (from increased aerosolization of TB by an influenza-co-infected adult) and enhanced risk of developing primary TB infection (due to influenza-induced immunomodulation). In older children, this immunomodulation could precipitate reactivation of TB infection (130). However, results from a single study comparing anti-influenza antibody titres in adults without and without pulmonary TB were inconclusive, and further studies, including in children, are warranted (131).

Mouse studies support the hypothesis that influenza virus induces immunological changes which may reduce the host's ability to contain TB infection, as has been observed for secondary bacterial infections (132). More rapid proliferation of mycobacteria and impaired mycobacterial-specific T cell responses are observed in mice co-infected with BCG and influenza compared to mice infected with BCG alone (133). In mouse models of *M. tuberculosis* infection, influenza co-infection enhances mycobacterial growth, (134, 135) through a type I IFN-signaling pathway. Prior exposure of mice to influenza type A before infection with *M. tuberculosis* also leads to enhanced mycobacterial growth and decreased survival. Influenza, like many viruses, induces a strong type I IFN response in humans. Influenza infection also downregulates certain TLRs, resulting in reduced neutrophil recruitment (136).

### Other Respiratory Viruses

Non-influenza respiratory viruses account for an even greater burden of morbidity and mortality in children than influenza. Respiratory syncytial virus is the most important, causing an estimated 33 million new episodes of acute LRTI per year in children age 5 years and under, 3.2 million of which necessitate hospital admission (137). The literature on non-influenza respiratory viral co-infection and TB is very limited. An Tanzanian study reported no difference in the frequency of observed influenza and non-influenza respiratory viral infections in adults with and without pulmonary TB (138). A South African study comparing children with “definite,” “unconfirmed,” and “unlikely TB” found no clear association between TB categorization and detection of specific respiratory pathogens (139).

## Respiratory Bacteria

Although less frequent than viral infections, childhood bacterial respiratory infections are common, particularly in the developing world. *Streptococcus pneumoniae* is the most common respiratory pathogen, *Haemophilus influenzae* and *Staphylococcus aureus* are also important causes of pneumonia. There were an estimated 3.7 million episodes of severe pneumococcal disease and 340,000 episodes of severe Haemophilus influenza type b (Hib) infection globally in children in 2015, with the highest incidences observed in Africa, South East Asia and Western Pacific (140).

Innate immune responses play a pivotal role in early host defense against extracellular bacteria including *S. pneumoniae* and *H. influenzae*. Bacteria are initially recognized by pattern recognition receptors consisting of TLRs, the cytosolic NOD-like receptors and DNA sensors. Recognition by these pattern recognition receptors triggers the release of pro-inflammatory mediators and stimulates the recruitment and activation of phagocytic cells. The resulting innate immune response involves complement (particularly C3), acute phase proteins (e.g., C-reactive protein, serum amyloid protein), neutrophils, macrophages, NK cells, and pro-inflammatory cytokines (TNF-α, IL-1, -6, -12, -17, -18) (141). Type I IFNs also appear to have an immunoregulatory role (142). Adaptive immunity is also important, particularly the synthesis of IgM, IgA, and IgG antibodies by B cells activated by bacterial antigens (143).

Several studies report on pneumonia and TB in children. In studies of pediatric pneumonia in TB-endemic countries, TB was diagnosed in 1.8–23% of cases and HIV co-infection was common (144–147). It is often challenging to determine what proportion of these cases represent TB pneumonia and what proportion are TB cases complicated by bacterial co-infection. A study of South African children aged 5 years and under with severe LRTI, found that 10% of HIV-infected and uninfected children with culture-proven TB had concurrent bacteraemia (148). Autopsy studies in children suggest that bacterial co-infections are important causes of death in children with TB (149–151). A recent study by Shimazaki et al. in the Philippines found that 29% of HIV-uninfected adults with pulmonary TB had purulent sputum with detectable bacterial DNA, most commonly *H. influenzae* (21.2%) and *S. pneumonia* (7.9%) (152). Bacterial co-infection was associated with an increased risk of 2 week mortality among confirmed TB cases.

These studies suggest that TB-bacterial pneumonia co-infection may be common in TB-endemic areas but there are few studies which investigate a possible interaction between them and the evidence is mixed. A systematic review of pneumonia in children from TB endemic countries suggested that TB might increase the risk of secondary bacterial pneumonia (149). On the other hand, a Tanzanian study which compared smear positive adult TB patients and household contact uninfected controls, found that respiratory bacteria were less frequently detected in the nasopharyngeal swabs of TB patients compared to controls. TB disease severity was higher only in those in whom both viruses and bacteria were detected (138). Evidence from vaccination studies is also mixed as described below.

## Other Important Bacteria

Most of the existing literature on TB-bacterial co-infection focuses on pneumonia-related bacteria. However, bacterial bloodstream infection (particularly *Salmonella enterica serovar Typhi, Staphylcoccus aureus*, and enterobacteriaceae), as well as bacterial zoonosis (brucellosis, leptospirosis, Q fever, and rickettsiosis) have also been reported as a common cause of febrile illness in children, particularly among hospitalized patients (153). Immunity to intracellular bacterial infections (ICBIs) such as salmonella, listeria or chlamydia, is mediated by host responses similar to those observed in *M. tuberculosis* infection. ICBIs are characterized by their ability to survive within macrophages and elicit a cell mediated response, stimulating CD4+ and CD8+ T cells through expression of the antigen epitope associated to either MHC class II or MHC class I, respectively. Activation of CD4+ T helper cells, specifically Th1 cells, leads to the secretion of IFN-γ which stimulates killing mechanisms inside the infected macrophage, liberating ICBI antigen epitopes, and increasing antigen presentation by bystander dendritic cells. It seems possible therefore that ICBIs could impact the immune response to TB. Case reports of co-infections with TB and ICBIs are present in the literature (154–157). However, an American population-based study found no difference in the risk of the ICBIs *Salmonella spp, Yersinia spp, and Listeria monocytogenes* in persons who developed TB vs. those in the general population, and found there were actually fewer *Chlamydia trachomatis* infections observed within the first year post-TB diagnosis compared to the non-TB population. Extrapulmonary TB was linked to a higher rate of salmonella infections compared to pulmonary TB but all 8 patients with salmonellosis and TB were also HIV co-infected (158).

Besides ICBI, multiple other bacteria have been reported to be associated with TB, including *S. aureus, Streptococcus milleri*, (159) enterococcus and klebsiella (160). Besides presenting in the lung, these concurrent tuberculous and bacterial infections have been described in other anatomical locations in children including the retropharynx (161) and central nervous system (162, 163).

## Non-tuberculous Mycobacteria

Non-tuberculous mycobacteria (NTM) include many environmental mycobacterial species other than *M. tuberculosis* and *M. leprae*. NTM and *M. tuberculosis* share microbiological attributes, induce similar immune responses, and have overlapping clinical manifestations (164). However, unlike *M. tuberculosis*, NTM are not always pathogenic and a major challenge with the diagnosis and management of NTM is to differentiate environmental contamination, colonization, and disease.

In the absence of primary or secondary immunodeficiencies, the host immune system is usually capable of containing and possibly eradicating NTM via established innate and acquired immune mechanisms. As for TB, containment of NTM relies on the integrity of the helper T cell type 1-cytokine pathway and cellular immune mechanisms. NTM disease has been associated with cystic fibrosis, mendelian susceptibility to mycobacterial disease and HIV infection. However, while the two latter are also risk factors of TB, there exists an inverse correlation between cystic fibrosis carriership and TB incidence, suggesting a lower susceptibility of cystic fibrosis carriers to TB (165). Exposure to NTM strains in the environment is thought to contribute to the variability of BCG vaccine, although the relationship is complex and unproven; both blocking and masking mechanisms have been proposed.

The epidemiology of NTM varies by world region (166). Attempts to understand the burden of NTM disease and identify risk factors in the pediatric population are hampered by inadequate mandatory NTM reporting and by the overlap of clinical presentation with TB. An association between increased disease incidence of mycobacterial disease caused by NTM and decreased incidence of TB has been suggested in adults although no causal relation has been proven. No estimates exist for the incidence of clinical syndromes caused by NTM in children or adults, and the available data is usually a by-product of studies assessing the burden of pulmonary TB in children, from whom NTM were isolated from respiratory specimens. The rate of NTM isolation in high TB burden settings varies between countries; from 2.7–26.3%, and increasing age is associated with a higher proportion (167–170). *Mycobacterium avium* complex species, *M. fortuitum, scrofulaceum*, and *gordonae*, are among the most frequently identified NTM in these studies.

Although concurrent TB and NTM is thought to be common, only a few reports have addressed this issue, (171–174) and the prevalence of co-infection depends on the sensitivity of the assay in detecting multiple species. In a recent study of HIV-infected children from African and South East Asian settings, NTM was isolated in 46/427(10.8%) of children, including 5 (1.2%) with both NTM and *M. tuberculosis* (170).

Whether or not concurrent infection is identified, the clinical significance of an NTM isolate in a patient receiving TB treatment is unknown (174, 175). Even if the isolate is likely to be clinically insignificant, it is plausible that co-infection plays a part in TB pathogenesis or time to sputum clearance (172). In adults, a previous history of TB disease is a risk factor for NTM pulmonary disease, probably due to structural damage to the lung (such as in bronchiectasis) altering mucociliary clearance and thereby predisposing the lung tissue to NTM isolation and disease (176, 177). Although the same phenomenon has not been described in children, bronchiectasis is often seen in children with HIV and chronic lung disease, (178) past TB, recurrent pneumonia, severe immunosuppression, and lymphoid interstitial pneumonitis (179).

## Leprosy

Leprosy, a disease caused by *M. leprae* has similar geographic endemicity as TB. A total of 14 adult co-infected cases have been published to date (180). There is no clear consensus on whether prior exposure to one mycobacteria offers protection or predisposition to the other, but several authors have suggested that impaired cell mediated immunity in patients with multibacillary leprosy may predispose them to TB co-infection (181, 182).

## Fungi

Mycobacteria share several features with pathogenic fungi including infection site, metabolic features, the composition and display of cell surface molecules, the range of innate immune receptors engaged during infection, and the ability to form granulomas (183). A number of immunodeficiencies, including chronic granulomatous disease, T cell disorders and deficiencies in the IL-17 pathways lead to increased susceptibility to mycobacterial and fungal infections, suggesting their immunopathogenesis is similar (184, 185). The innate immune response is defined by an array of pattern recognition receptors, such as the TLRs, which recognize bacterial targets such as lipopolysaccharide, flagellin etc. *M. tuberculosis* and fungi are both “poor agonists” of the TLRs, and virulent strains of *M. tuberculosis* have even been reported to down-regulate MyD88, a key TLR signaling molecule (186). Virulent strains of both mycobacteria and fungi appear to induce a Th2 rather than a Th1 response in order to evade the host immune response. The key immunopathological feature of TB is the formation of granuloma in the lungs—a condition that can be considered mutually beneficial for host and pathogen, as it constrains the pathogen, while providing a microenvironment for replication of the organism. Granuloma are also noted in fungal, but not other bacterial infections. Both mycobacterial and fungal disease dissemination depends on the ability of the host to maintain the granuloma, a balance influenced both by virulence factors of the invading organism, as well as a variety of external factors including age, malnutrition, and co-infections.

Mycobacterial-fungal co-infection most frequently occurs in the context of immunodeficiency, such as HIV, bone marrow transplant, and primary immunodeficiencies, such as severe combined immunodeficiency or chronic granulomatous disease. *Pneumocystis jirovecii* pneumonia (PCP) is one of the commonest opportunistic infections in HIV-infected patients in the developed world and although less common in low and middle income countries, still poses a threat. PCP-TB co-infection has been described in these patients, but not in HIV-uninfected populations (146).

A number of adult studies have suggested that TB-fungal co-infection with agents such as Candida and Aspergillus may occur in between 6.5 and 40% of cases of pulmonary TB (187, 188). Co-infection was more common in adult patients with multidrug-resistant-TB, who were more likely to have significant lung pathology. Chronic pulmonary aspergillosis affects patients without obvious immune compromise, but with concurrent or prior TB disease. Chronic pulmonary aspergillosis has recently been recognized as an important global health problem, associated with significant morbidity and mortality. The most common predisposing factor is previously-treated TB, independent of HIV infection (189). Although this is not seen frequently in children, identifying, and treating TB promptly in childhood may prevent chronic lung disease in adulthood.

## Microbiome and Gastrointestinal Infections

Between ten trillion and a hundred trillion organisms live within, or on the surface of, each human being, termed the human microbiota (190). The genetic material within these organisms is referred to as the human microbiome. Although the majority of the microbiome is within the gut, an important population resides in the lung and cross-talk between the two, termed the gut-lung axis, is emerging as a central concept in our understanding of the microbiome (191, 192). Bacteria, bacterial toxins, cytokines, metabolites, and hormones move between the two populations through the bloodstream, with the two communicating to each other and each influencing the composition of the other.

The microbiome can impact on host immunology in a number of ways (193). First, it can act as a barrier to the overgrowth of other organisms, through what has been termed colonization resistance. In this situation, competition for limited resources limits the growth of non-microbiota organisms. Second, it can impact on the innate immune system, both through the stimulation of TLRs to produce antimicrobial peptides (194) as well as through modulation of innate immune cell development. Finally, in addition to its impact on the innate system, the microbiota can also prime the adaptive immune system, particularly in its mucosal T-cell response.

Our understanding of the microbiome is rapidly progressing, and there is increasing interest in the role of the microbiome in TB pathogenesis, (195–197) and specific interest in the gut-lung axis (198, 199). While a comprehensive review of the microbiome and potential TB risk is beyond the scope of this article, it is possible that the microbiome impacts on risk of TB infection following exposure, risk of disease progression following infection and also disease outcome. Although there may be some mycobacterial exposure in the gut (mainly with *M. bovis*), most mucosal interaction with *M. tuberculosis* is within the lung. Wu et al. used 16S RNA sequencing to analyse and compare the sputum microbiota of adults with new TB, recurrent TB, TB treatment failure and healthy controls, demonstrating significant differences in the abundance of commensals between the groups (200).

The lung microbiota may influence the host response at the initial point of *M. tuberculosis* exposure, through colonization resistance and stimulation of TLR-mediated responses (201). However, as TB infection develops, stimulation of host immune cells and cytokines is required to contain proliferation of the mycobacteria, and it is likely that this is influenced by both the lung and gut microbiomes. It has been demonstrated that individuals infected with *H. pylori*, a bacteria that resides in the stomach of nearly 50% of the world's population, had higher TB antigen-induced IFN-γ responses, compared to those with negative *H. pylori* serology. Those with positive *H. pylori* serology were also less likely to progress to TB disease compared to those with negative serology (199).

The composition of the gut bacterial microbiome changes with age (202). Soon after birth the neonatal gut is dominated by *Enterobacteriaceae*. This is soon replaced by predominately *Bifidobacteria* which continue to be the most commonly represented class of bacteria until the child is weaned onto solids. Following weaning, the adult pattern is seen with *Bacteroides, Prevotella, Ruminococcus, Clostridium*, and *Veillonella* occupying the gut (203).

In addition to the alterations in the microbiota seen with age, the organisms that cause enteric infections, and frequency of infections, also changes (204). Infants experience infections with rotavirus, cryptosporidium, *E. coli, Shigella* and adenovirus most commonly. Children 12–23 months have fewer infections but with similar pathogens. Children over 2 years, have fewer infections still mainly due to *Shigella* and rotavirus (205). As rotavirus vaccination becomes more widespread, this distribution will likely change. The relationship between malnutrition, microbiome, intestinal infections, and host immunological status is complex as interplay exists between each of these and is beyond the scope of this review (206–209). It is likely that this combination will impact on TB pathogenesis.

## Helminths

Studies of the immune interactions between helminths and TB have largely focused on the effect of co-infection on: the efficacy of BCG vaccination; diagnostic tests for TB infection (TST and IGRA); and the role of anti-helminthic treatment on TB outcome, as measured by progression from TB infection to disease (210). Results of these studies have been variable, likely reflecting the diversity of environmental influences and possibly differing immune responses induced by different helminth species.

The most common helminth infections globally are the soil-transmitted intestinal helminths, including the roundworm *Ascaris lumbricoides*, the hookworms *Necator americanus* and *Ancylostoma duodenale*, the whipworm *Trichuris trichiura*, and *Enterobius vermicularis*, and *Strongyloides spp*. Tissue damage by these intestinal helminths during feeding and migration results in production of danger associated molecular patterns and induce an immune response characterized by IgE secretion, IgG4 production, eosinophilia, production of Th2 cytokines (IL-4, IL-9, IL-10, IL-13) and induction of FOXP3+ regulatory T cells and the associated regulatory cytokines Tumor Growth Factor-β and IL-10. As with measles, it is hypothesized that this shift away from Th1 responses is responsible for making individuals infected with helminths more susceptible to TB infection and disease. This regulation can affect not only responses to helminth antigens, but also responses to unrelated antigens such as TB or the BCG vaccine.

There is variability in studies of the influence of helminth infection on diagnostic tests for TB infection, mostly focusing on the TST. A recent South African study showed no impact of deworming on either the TST or IGRA in children (211) however Thomas and colleagues found an increase in indeterminate results of IGRA assays (212). The influence of helminth infection on progression to TB disease is similarly unclear. In one study on recent immigrants to the UK, those patients with helminth infection showed a significant increase in CD4+ FOXP3+ regulatory T cells compared to those without helminth infection. Following anti-helminth treatment, the frequency of regulatory T cells decreased, with an associated increase in IFN-γ producing CD4+ T cells, demonstrating a potential mechanism for susceptibility to TB disease (213). Although Indonesian children with helminth infections had similar frequencies of regulatory T cells in comparison with those without infections, *in vitro* T-cell functional studies demonstrated suppressed IFN-γ responses to whole blood stimulation with BCG and *Plasmodium falciparum*, an effect that was reversed with depletion of regulatory T cells (214). Furthermore, children with ascaris or schistosomiasis infections showed significantly increased Th2 responses to mycobacterial stimulation compared to uninfected children, and these responses persisted for up to 6 months following confirmed successful anti-helminth treatment in children with schistosomiasis, but not ascaris (215). In addition, these children had also evidence of epigenetic changes due to helminth infection, as measured by corresponding DNA methylation signatures.

However, no longitudinal studies to date have been powered to determine whether helminth infection influences progression from TB infection to disease, or to confirm a direct relationship between helminth infection and TB disease severity. While *in vitro* studies clearly demonstrate that pre-exposure or co-incident infection with filarial, hookworm, strongyloides and schistosoma infections is associated with downregulated Th1 and Th17 responses and upregulate Th2 and Regulatory T cells to mycobacterial antigens, (210, 213–215) these findings have not been replicated in patients with TB disease, although increased regulatory and Th2 responses have been shown in patients with asymptomatic helminth infections and TB disease (216). Adults from helminth endemic countries with TB disease demonstrate a mixed Th1/Th2 picture, with reduced CD8+ T cells, while those from low helminth settings have a Th1, IFN (type I and IFN-γ) predominant phenotype (217). In Brazil and Ethiopia, patients with TB disease were more likely to have intestinal helminths than those unaffected and co-infection was associated with more advanced TB disease (218, 219). However, a randomized control trial showed no benefit of anti-helminthic treatment on clinical improvement of TB (220). An Indian study of more than 5000 patients over 10 years showed no effect of helminth co-infection on progression to disease nor severity of disease in an adult population although as helminth status was only measured at baseline, it is difficult to draw definitive conclusions about this (221). In endemic regions, the burden of helminthic infection peaks by adolescence, suggesting at least partial protective immunity with increasing age. Larger longitudinal studies in young children could elucidate the impact of helminth co-infection on TB disease progression.

## Malaria

Malaria kills more individuals each year than any other parasitic disease, responsible for ~445,000 deaths in 2016, most of them young children in sub-Saharan Africa (9) Of the estimated 216 million cases of malaria worldwide in 2016, 90% took place in the WHO African Region, followed by the WHO South-East Asia region.

Protection against malaria is postulated to be mediated by both cell mediated and humoral immune responses. *P. falciparum* infection is characterized by the production of pro-inflammatory cytokines including IL-1β, IL-6, IL-12, TNF-α, IFN-γ, and associated Th1 cytokines (222). The variability in the TNF-α response to endotoxins could mediate differential host responses which contribute to severe malaria when TNF-α levels are high (223). High levels of IFN-γ have also been associated with severe malaria infection, but evidence is inconsistent (224). Moreover, malaria has several immunomodulating effects during acute infection including lymphopenia, decreased levels of CD4+ T cells, and a functional immunosuppression greater than can be attributed to the quantitative fall in CD4+ cells (225, 226).

Although the first published report of malaria TB co-infection was in 1945, (227) there are only few papers published to date, including a case of perinatal malaria and TB in an infant (228), and several epidemiological surveys with prevalence varying from 37% in hospitalized adult and pediatric TB patients in Angola, to 4.3% among adult pulmonary TB patients in Tanzania (229, 230). Co-infections have been studied in both animal models and humans (226, 231–234), and biological interactions seem to exist between *P. falciparum, M. tuberculosis*, and a shared human host.

Malarial parasites decrease the host's effective humoral and cellular immune responses to *M. tuberculosis*, and in experimental models co-infection exacerbates acute and chronic mycobacterial infection (235, 236). In humans, co-infection with malaria and TB seems to modulate the immune response to confer immunological protection against malaria while weakening response to TB (231). Chukwuanukwu et al. recently showed that patients co-infected with malaria had an increase in the production of Th2-associated cytokine IL-4 and anti-inflammatory IL-10 in tuberculin-stimulated cells of TB patients >12 years of age compared to malaria-free TB patients, suggesting that malaria co-infection diverts immune response toward a Th2/anti-inflammatory response (231). However, the true impact on risk of infection and disease progression remains unclear. Some authors suggest that TB co-infection has no impact on the outcome of induced experimental cerebral malaria in mice and attribute this to the induction of the inflammatory response which rapidly dominates (233).

Malaria may also impact the ability to diagnose TB infection. Drabe et al. evaluated the performance of IGRA and IP-10 release assays in adult patients with concurrent malaria in Tanzania and found that during malaria infection, IP-10 and IFN-γ levels in the unstimulated samples were elevated, mitogen responsiveness was impaired and CD4 cell counts were decreased (225). These alterations reverted rapidly after malaria treatment.

In terms of the impact of *M. tuberculosis* on malaria immunity, animal models suggest that *M. tuberculosis* offers some non-specific protection against rodent plasmodium, reflected by reduced parasitaemia due to mycobacterium-induced pro-inflammatory response (IFN-γ and TNF-α mediated activation of macrophages) (235, 237).

Beyond the complex immunological interactions described above, both diseases seem to share common pathogenesis pathways and genetic factors affecting susceptibility. First, matrix metalloproteinases (MMP), a family of proteolytic enzymes involved in modulating inflammatory response, disrupting tight junctions and degrading sub-endothelial basal lamina, seem to play a critical role in both TB and cerebral malaria pathogenesis (238–240). Data from *in vitro* and *in vivo* studies suggest that MMP might be involved in the pathogenesis of cerebral malaria through blood brain barrier damage and leakage as well as through induced inflammatory response. In addition, MMP-1 and MMP-9, as well as other MMP, have been implicated in lung matrix destruction in TB through degradation of fibrillar collagens and other matrix components. MMP-9 concentrations are also increased in the cerebrospinal fluid of patients with TB meningitis and correlated with extent of neurological compromise (238). Moreover, it has recently been suggested that, through complement-mediated lysis, α-Gal immunity might protect against malaria, TB, as well as other NTM, leishmania and trypanosoma; all of which express α-Gal on their surface (241, 242). Although the direct association between blood type, low α-Gal antibody titres and susceptibility to pathogens containing α-Gal still remains to be verified, Cabezas-Cruz et al. suggest that blood type B decreases the anti-α-Gal antibody levels increasing the risk of malaria or TB (242). It has been shown that the incidence of blood type B is positively correlated with the incidence of malaria and TB, but not dengue, which does not produce α-Gal antigen.

## Other Protozoa

Besides malaria, multiple other protozoa, including leishmania, trichomona, toxoplasma, giardia and entamoeba, have been described in co-infection with TB although the literature is limited to several case reports, few epidemiological surveys and some immunological research (236). Among non-malaria protozoa, leishmaniasis, a vector borne zoonosis caused by an intracellular pathogen, is reported to co-exist more frequently with TB. In Sudan, up to 77% of TB patients (adult and children) were positive for the leishmania skin test in the community (243). In a systematic review of TB and parasites co-infection published in 2013, the authors found that TB and parasitic diseases were reported as risk factors for each other (236). Two studies conducted in Sudan and Ethiopia evaluated the inter-relationship of TB and parasitic diseases and reported that the risk of TB was higher in patients who were *Leishmania donovani* positive or *Giardia lamblia* positive (243, 244). Likewise, TB patients were more easily infected by *Leishmania donovani* and *Giardia lamblia* than those without pulmonary TB.

Several reports (245–247), including a pediatric case (248), have described the potential immunological changes observed during co-infection of leishmania and TB, the development of both depends on impaired cell-mediated immunity. A case report on *the triple infection of leishmania, M. tuberculosis*, and *M. lepra* showed that the adult patient, with no recognized immunodeficiency, was unable to mount a Th-cell response to upregulate the IL-12 receptor expression after stimulation of the triple infection (245). It has recently been suggested that TB could be a decisive contributing factor to the high mortality observed in patients with visceral leishmaniasis and HIV, although the authors conclude that the true prevalence and impact of co-infection remain unclear (246).

## Vaccinations

As discussed above, infants, and young children are highly susceptible to both TB infection and disease and we have described how a variety of childhood infections, many of which are most common in the early years, may impact on immune responses essential to protection from TB. During these early years, infants also receive several routine vaccinations, both live and inactivated, that may also have an immunomodulatory effect on TB susceptibility (Table 2).

**Table 2:** Type of vaccines and how they may influence susceptibility and protection to tuberculosis in children.

**Type of vaccine**	**Examples of vaccine**	Immune responses^*^	**Possible influence on TB immunity**
Live attenuated vaccine	Measles, mumps Rubella (MMR) Rotavirus Varicella Yellow Fever BCG	Induce cytotoxic T cells CD4 and CD8 Heterologous immune responses, in particular BCG	Cytotoxic T cells more IFN—greater anti-TB immune responses Possible mechanism for protection against disseminated BCG
Inactivated vaccine	Hepatitis Influenza A Inactivated polio	Th2 stimulation IL2, IL4, IL5, IL6 B cell activation, Memory B cell IgM to IgG class switch	Th2 predominant—less effective anti-TB immune response
Toxoid vaccines	Diphtheria Tetanus	Th2 stimulation B cell activation Memory B cell IgM to IgG class switch	Th2 predominant—less effective anti-TB immune response
Subunit/conjugate	Haemophilus influenza b (Hib) Pneumococcus Meningococcal B & C Hepatitis B Human papillomavirus	Polysaccheride vaccines—T cell independent response, no Th2 Conjugate vaccines—T cell dependent Th2, more specific than toxoid	Th2 predominant—less effective anti-TB immune response

* *Not complete list of immune responses, describing commonest, or predominant immune response*.

Antigen specific induction of T and B lymphocytes and the “adaptive” immune system have traditionally been ascribed the role of vaccine-induced protection. However, cells in the innate immune system, such as monocytes, macrophages, dendritic cells, and NK cells, appear to be influenced by contact with a variety of antigens, leading to functional reprogramming, which facilitates rapid, enhanced responses to future, non-specific threats—termed “trained immunity.” The duration and mechanisms by which this long-term innate immunity is induced are the subject of extensive research (249).

BCG is a live, attenuated vaccine that is widely administered to infants in most areas endemic for TB and although it provides imperfect protection against TB infection and disease, it does result in at least partial protection against severe manifestations of TB disease during the first years of life (250, 251). The BCG vaccine unfortunately does not protect all infants from dissemination of *M. tuberculosis*, and why it works for some and not for others is still not sufficiently explained through immune mechanisms (252). Recently, in a study of adult BCG vaccinated healthy volunteers, cytokines associated with trained immunity, TNF-α, IL-1β, and IL-6 were found to be induced, and associated with control of mycobacterial growth in an inhibition assay (253). Novel markers of trained immunity, CXCL-9 and CXCL-10 were also identified in this study. Both helminth infection and NTM infection have been implicated in variable BCG responses, although the evidence is variable and no definitive study has confirmed a mechanism to date.

Most efficacious vaccines induce antibody mediated protection, and although there is some evidence that antibody responses may mediate some protection against TB, this has not been explored, or understood to the same extent as cell-mediated responses (254, 255). Recently, total IgG levels were found to be protective against TB infection as measured by IGRA positivity in infants. There was a trend toward a protective effect of BCG and measles IgG to Quantiferon positivity, and the authors concluded that BCG and measles vaccination may provide heterologous protection against *M. tuberculosis* infection, however this is speculative, and numbers were small. Conversely, as mentioned above, measles vaccination has previously been associated with hypo-responsiveness to the TST (256, 257).

A relationship between *S. pneumoniae* and TB has been explored. In a clinical trial of a 9-valent polysaccharide pneumococcal vaccine, South African children who received the vaccine were 43% less likely to be hospitalized with culture-confirmed pulmonary TB than those who received placebo. In HIV-infected children, vaccines were 47% less likely to be diagnosed with culture-confirmed pulmonary TB compared to placebo recipients. These observations were attributed to pneumococcal infections precipitating hospitalisations and diagnosis of pulmonary TB (258). However, a subsequent study by the same group looking at the trends in pediatric pulmonary TB hospitalisations found no evidence for an effect of the vaccine (259). Limited studies exist on whether other vaccines may increase or decrease the susceptibility of infants and young children to TB and further studies are warranted.

## Research Priorities

A great deal of further work is required to better understand the relationship between TB and other infections. We need more details of the epidemiology of co-infections to understand how commonly they occur and in which patient populations they take place. We need to know more about how co-infections impact on a child's immune system, specifically in relation to the way that the host responds to *M. tuberculosis*, and also how this changes with age. A more comprehensive understanding of the effect of co-infections on the diagnosis and treatment of children with TB would help manage children with both illnesses and finally it would be useful to understand how vaccination strategies for co-infections might impact on the pathogenesis of TB in children. We outline some of these issues in Table 3.

**Table 3:** Potential study designs to answer the priority research questions in the field of tuberculosis co-infections.

**Type of research**	**Research question**	**Research design**
Basic science research	Does co-infection influence mycobacterial immune responses?	Evaluate interaction between host and *M. tuberculosis* by stimulating blood from children infected with various pathogens to *M. tuberculosis* in the laboratory *In vitro* assessment of immunological impact of co-infection of blood with variety of pathogens and mycobacteria *in vitro* Impact on novel diagnostics based on host transcriptomics. Presumed altered results if different co-infections—need to include diagnostic tests for co-infection as part of these studies
Surveys of disease prevalence	Describe the epidemiology of TB-pathogen co-infection in a variety of geographical areas Describe the impact of other environmental factors/interventions on TB exposure outcomes	Quantify how common co-infections are in children of different ages, in different geographical areas and evaluate in: (a) all children; (b) children exposed to TB; (c) children with TB infection; (d) children with TB disease. These studies would also ideally collect samples for serological analyses, transcriptomics, presences of pathogen particles in blood (antigen/PCR) and culture samples from blood or other sites Determine the effect of malnutrition (worsening of microbiome and associated with helminth), HIV, and other comorbidities on the immune response to TB
Longitudinal cohorts	Describe the natural history of TB exposure in children following a variety of different exposures/interventions	Mother -infant and birth cohort studies—exploring influence of co-infections on mycobacterial responses or outcomes following household TB exposure TB natural history studies—observational and interventional cohorts taking part in MDR-TB chemoprophylaxis studies Infants born to mothers taking part in maternal vaccination studies—these infants will be having samples taken to explore the impact of maternal immunization on their vaccine responses, need to gather data on co-infections, TB exposure and outcome as part of these studies
Vaccine cohorts	Do co-infections affect efficacy or immunogenicity of novel vaccine candidates	Explore influence of co-infections on pediatric and adult cohorts taking part in novel vaccine studies. These studies would also ideally collect samples for serological analyses, transcriptomics, presences of pathogen particles in blood (antigen/PCR), and culture samples from blood or other sites and analyse the impact of various pathogens isolated
Direct studies of co-infection	Do interventions to change co-infections alter outcome following TB exposure	Evaluation of host directed therapies to mitigate effects of co-infections. Evaluation of novel vaccine candidates, or booster doses of BCG to mitigate effects of co-infections Explore the role of vaccines against specific pathogens in protecting against TB—e.g., measles, CMV, Influenza, etc.
Programmatic changes	Can optimisation of current programmes improve TB outcomes	PMTCT—great success, what can we do to improve it, and improved access to ARV in those who are infected, in particular young women who may become pregnant Impact of interventions which may influence early childhood illnesses impact on the risk and outcomes of TB (i.e., promotion of maternal health and antenatal care, which may in turn influence microbiome, birth weight, maternal antibody transfer, and protection)

## Conclusions

This review suggests that for many children in low resource settings co-infections are common and it is likely that many childhood illnesses impact on the host response to *M. tuberculosis*, affecting risk of TB infection and disease. Vaccinations are also important, both for direct protection from the pathogens vaccines are designed for, but also for the indirect protection from vaccines such as BCG and measles. Better understanding the relationship between co-infections and TB may allow clinicians to improve the care of children at every stage of TB pathogenesis, through early treatment of co-infections, immunomodulation, or vaccination.

## Author Contributions

EW and JS devised the manuscript. EW, EL-V, CB, and JS contributed to the manuscript.

### Conflict of Interest Statement

The authors declare that the research was conducted in the absence of any commercial or financial relationships that could be construed as a potential conflict of interest.
